# Autologous Blood Injections in Temporomandibular Hypermobility: A Systematic Review

**DOI:** 10.3390/jcm12175590

**Published:** 2023-08-27

**Authors:** Maciej Chęciński, Kamila Chęcińska, Iwona Rąpalska, Natalia Turosz, Dariusz Chlubek, Maciej Sikora

**Affiliations:** 1 Department of Oral Surgery, Preventive Medicine Center, Komorowskiego 12, 30-106 Kraków, Poland; maciej@checinscy.pl (M.C.); iwona.rzasa@uj.edu.pl (I.R.); 2 Department of Glass Technology and Amorphous Coatings, Faculty of Materials Science and Ceramics, AGH University of Science and Technology, Mickiewicza 30, 30-059 Kraków, Poland; checinska@agh.edu.pl; 3 Department of Oral Surgery, Jagiellonian University Medical College, Montelupich 4, 31-155 Kraków, Poland; 4 Institute of Public Health, Jagiellonian University Edical College, Skawińska 8, 31-066 Kraków, Poland; natalia.turosz@gmail.com; 5 Department of Biochemistry and Medical Chemistry, Pomeranian Medical University, Powstańców Wielkopolskich 72, 70-111 Szczecin, Poland; sikora-maciej@wp.pl; 6 Department of Maxillofacial Surgery, Hospital of the Ministry of Interior, Wojska Polskiego 51, 25-375 Kielce, Poland

**Keywords:** temporomandibular joint, temporomandibular disorders, intra-articular injections, blood

## Abstract

The injection of autologous blood (AB) is one of the methods of treatment of recurrent dislocations in the temporomandibular joints (TMJs). Due to the low invasiveness of this technique, it is reasonable to evaluate it in accordance with the standards of evidence-based medicine. The purpose of this systematic review is to identify primary studies on AB injection for the treatment of TMJ hypermobility and assess the therapy for effectiveness. This systematic review was conducted in accordance with the current “Preferred Reporting Items for Systematic Reviews and Meta-Analyses” guidelines. Controlled randomized trials comparing dislocation episode rates, range of motion in the TMJ, or articular pain intensity were adopted as the eligibility criteria. Final searches were conducted on 11 June 2023 using Bielefeld Academic Search Engine, Elsevier Scopus, and the National Library of Medicine: PubMed. Trials were assessed using the “Oxford Center for Evidence-Based Medicine 2011 Levels of Evidence” scale and “A revised Cochrane risk-of-bias tool for randomized trials”. The results of the individual studies were tabulated, syntheses were illustrated in graphs. Twenty two studies involving 982 patients were included in the qualitative analysis, of which seven studies involving 390 patients were subject to quantitative analysis. None of the included randomized controlled trials presented a high risk of bias, 75% of them raised some concerns. In a three-month observation, administration of AB was more efficient in limiting temporomandibular dislocations than hypertonic dextrose (1 study, 32 patients, relative risk = 0.33, odds ratio = 0.29) and no difference in outcomes was observed between intracavitary and pericapsular administration compared to pericapsular injection alone (2 studies, 70 patients, relative risk = 1.00, odds ratio = 1.00). Injections of AB into the temporomandibular joints are effective in preventing further TMJ dislocation episodes in 75–94% of patients. This study received no funding.

## 1. Introduction

### 1.1. Background

The paired temporomandibular joints (TMJs) are responsible for moving the mandible. The range of motion in each TMJ is limited by the shape of the mandibular fossa and articular tubercle as well as the ligament apparatus. The joint cavity is surrounded by a joint capsule, which loosens in cases of mandibular hypermobility. Subluxation or luxation of the TMJ consists of the mandibular head with the articular disc crossing the largest convexity of the articular tubercle [1]. The second diagnosis is made when repositioning requires qualified medical assistance [1]. Recurrent dislocation significantly affects the patient’s quality of life and requires treatment leading to a cure [2].

The dominant therapeutic strategies are counteracting or reducing the strength of the mandibular abductor muscles; diminution in the elasticity of the ligament apparatus; obstruction of the mandibular head path; clearing the way for the head of the mandible (to facilitate repositioning of the dislocation); and replacement of the joint with a prosthesis. In cases of one-off or rarely recurrent dislocations, repositioning and immobilization are used. Both external stabilization of the chin, e.g., with an elastic bandage or a cervical collar, and intraoral elastic fixation with dental or bone anchorage are applicable [3,4]. The weakening of muscle strength is achieved by injecting the lateral pterygoid muscle with botulinum neurotoxin [4,5,6]. The elasticity of the ligaments of the joint capsule and the retrodiscal zone is reduced by administering an irritant, which is referred to as prolotherapy [7,8,9]. Limiting the movements of the mandibular head is achieved by soft tissue surgery (joint capsule plication, temporalis muscle scarification, lateral pterygoid muscle plasty), osteoplasty (articular eminence augmentation, zygomatic arch down-fracture), or alloplastic material insertion (screws, mini-plates, etc.) [10]. The opposite approach is to abolish joint eminence, which clears the path for dislocation reduction [10,11]. Attempts are made to perform some of the above-mentioned surgical procedures using the endoscopic technique, including retrodiscal tissues cauterization, capsulorrhaphy, and eminoplasty [10]. 

In general, (1) non-invasive methods are of little use in cases of habitual dislocations, (2) arthroscopy is quite invasive, less accessible, relatively expensive and difficult to master, and (3) open surgery is highly invasive. Compared to them, minimally invasive techniques deserve attention, as they allow for limiting surgical access to a single puncture or a series of punctures. Such protocols consist of intra- and peri-articular injection of hypertonic dextrose or autologous blood (AB) into and around the TMJs cavities [7,8,9]. The latter method of treatment creates conditions within the joint similar to blood extravasation, albeit without injury [12]. This leads to a reduction in the range of motion of the mandible, but not to ankylosis [1,13,14,15]. Despite the promising results of the administration of AB into TMJs, it is necessary to verify and validate these outcomes in the light of evidence-based medicine.

### 1.2. Rationale

Seven systematically conducted reviews covering intra-articular injections of AB in the treatment of temporomandibular hypermobility have been published so far [13,16,17,18,19,20,21]. In 2015, promising results of the discussed technique were first indicated based on four prospective clinical trials [16]. Prechel et al. and Renapurkar et al.’s reviews suggest that in recurrent temporomandibular dislocations, injection treatment is the first choice, supplemented by open surgery when ineffective [17,18]. In 2020, based on two randomized controlled trials, AB injections were the best-documented treatment technique for recurrent TMJs dislocations [19]. A current systematic map of temporomandibular intra-articular injections identified eleven clinical trials (including four randomized) on administering self-derived blood into joint cavities [21]. The proven effectiveness of the technique and the greater body of evidence than summarized so far justify a systematic review of AB injection for the treatment of TMJ hypermobility.

### 1.3. Objectives

The purpose of this systematic review is to identify primary studies on AB injection for the treatment of TMJ hypermobility and assess the therapy for effectiveness.

## 2. Materials and Methods

### 2.1. Eligibility Criteria

Primary clinical trials for the treatment of mandibular hypermobility with injections of unprocessed blood were included in the review. Randomized controlled, non-randomized controlled, and uncontrolled studies were allowed. Of these, controlled randomized trials comparing dislocation episode rates, range of motion in the TMJ, or articular pain intensity were promoted for quantitative analysis. Detailed eligibility criteria are presented in Table 1.

### 2.2. Information Sources

Final searches were conducted on 11 June 2023 using Bielefeld Academic Search Engine (over 300 million records), Elsevier Scopus (over 91 million records), and the National Library of Medicine: PubMed (over 35 million records) [22,23,24].

### 2.3. Search Strategy 

The following query was entered into each of the search engines: temporomandibular AND blood AND (intra-articular OR intraarticular OR intra-articularly OR intraarticularly OR intra-cavitary OR intracavitary OR injection OR injections OR injected OR puncture OR punctures OR administration OR administrations OR administered)

### 2.4. Selection and Data Collection Process

The selection was performed in line with the “Preferred Reporting Items for Systematic reviews and Meta-Analyzes” methodology [25]. The process was improved by use of the Rayyan automation tool (Qatar Computing Research Institute, Doha, Qatar and Rayyan Systems, Cambridge, MA, USA) [26]. The individual selection stages were as follows: (1) duplicate identical records were automatically rejected; (2) each of the potential duplicates identified by the software was evaluated; (3) abstracts with software-indicated keywords for potential inclusion and exclusion were individually screened; (4) the remaining records were manually assessed in full-text, which was followed by data collection (M.C., K.C.). It was decided that in case of a discrepancy between the judges’ assessments in stages 2 or 3, the record would be promoted further. Disagreements in the full-text evaluation and data extraction could be discussed until a consensus was reached.

### 2.5. Data Items 

The following data were extracted for the study group and control group(s): (1) number of patients; (2) administered substance; (3) deposition sites; (4) injectable volume; (5) additional interventions; (6) number of intervention repetitions; (7) frequency or presence of dislocation episodes before intervention and during follow-up; (8) range of mandibular mobility before treatment and during observation; (9) initial and subsequent articular pain levels (in visual analog scale).

### 2.6. Study Risk of Bias Assessment 

All clinical trials were assessed using the “Oxford Center for Evidence-Based Medicine 2011 Levels of Evidence” scale [27]. Bias risk assessment in randomized trials was performed using “A revised Cochrane risk-of-bias tool for randomized trials” [28]. Studies with no high risk of bias in either domain were included in the quantitative analysis.

### 2.7. Effect Measures and Synthesis Methods

Continuous outcome measures were tabulated in raw and unified values (transformed to percentage of baseline values). In the latter form, they were additionally synthesized in charts whenever data before and after the intervention were available. Data were also dichotomized into (a) improvement and (b) no improvement for the purpose of measuring the effect with the risk ratio (RR) and odds ratio (OR) [25].

## 3. Results

### 3.1. Study Selection

**Figure 1:**
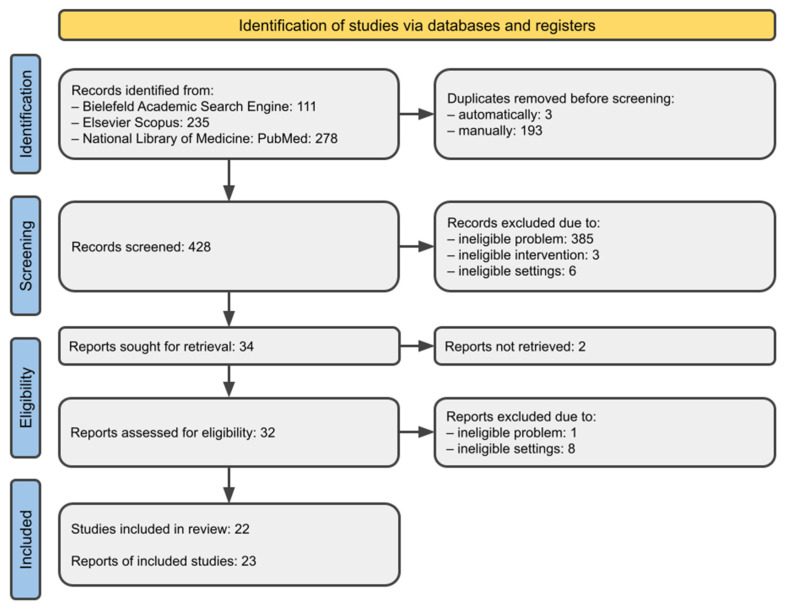
Study selection.

Of the 624 records identified, a total of 196 duplicates and 394 ineligible screened items were rejected. Six single case reports, one series of three cases, two not retrieved papers from 1981, one animal study, and one clinical study protocol were rejected at the full-text evaluation phase [29,30,31,32,33,34,35,36,37,38,39]. Detailed selection steps are illustrated in Figure 1.

### 3.2. Study Characteristics

Of the 22 studies included in the review, 10 were controlled, including 8 randomized. In each study group, AB was deposited both into the joint cavity and pericapsular tissues. The number of administrations varied between studies and where ranges are given, this means administration until a satisfactory result, patient withdrawal, or the end of the study. Detailed characteristics of the studies are presented in Table 2.

### 3.3. Risk of Bias in Studies

**Figure 2:**
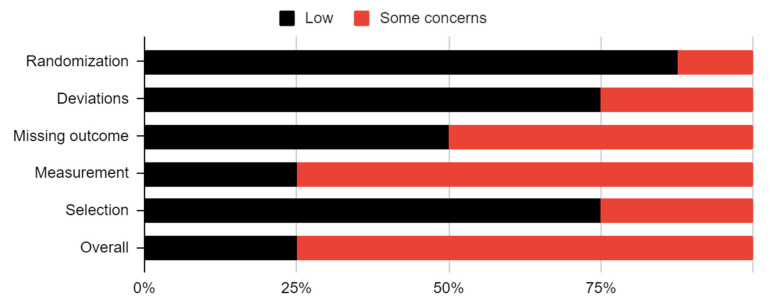
Risk of bias in studies.

Eight studies with a level of evidence of 2 were qualified for assessment of the risk of bias. There was no high risk of bias for any study in any domain (Table 3, Figure 2). Therefore, all randomized controlled trials were promoted for outcomes extraction.

### 3.4. Results of Individual Studies

Population data and outcomes of the studies included in the synthesis and are summarized in Table 4. Changes in the mandibular abduction range were the only variables reported in most studies. In addition to the variables collected for the purposes of this review, some authors reported the presence of acoustic symptoms, patient satisfaction, and craniomandibular index scores. The time-varying number of patients presenting episodes of temporomandibular dislocation, the extent of mandibular abduction, and visual analog scale of articular pain values are summarized in Table 5, Table 6 and Table 7.

### 3.5. Results of Syntheses

**Figure 3:**
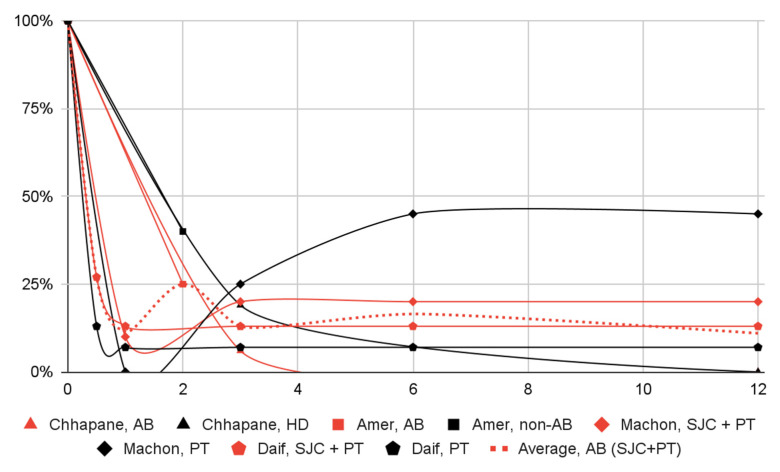
Change over time (in months) in the proportion of patients with dislocation episodes (percentage of baseline) by patient group.

**Figure 4:**
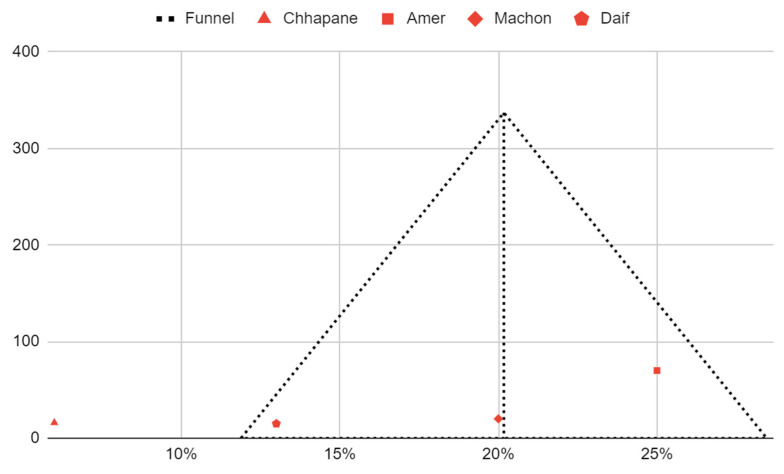
Funnel plot illustrating the number of patients and the proportion of patients with dislocation episodes (percentage of baseline) in 2–3 months of the groups receiving intra- and pericapsular autologous blood injections.

**Figure 5:**
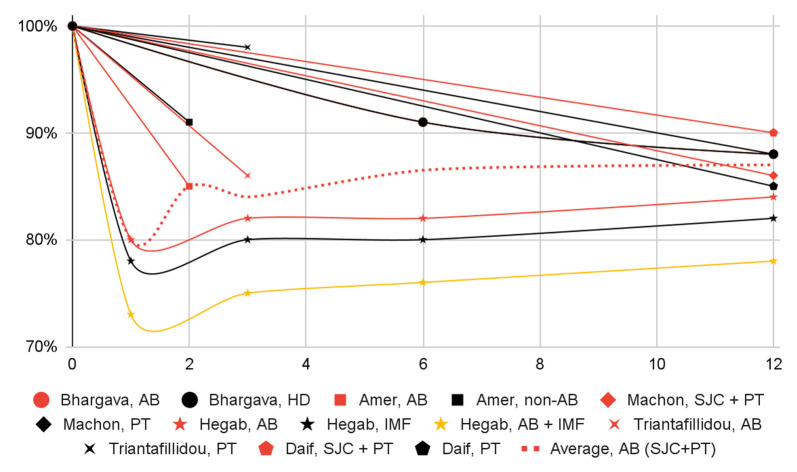
Change over time (in months) of mandibular abduction (percentage of baseline) by patient group. Entire Bhargava, AB and Bhargava, HD graphs overlap (Bhargava, HD visible). Bhargava, AB and Bhargava, HD, and Machon, PT start and end points overlap (Bhargava, HD visible).

**Figure 6:**
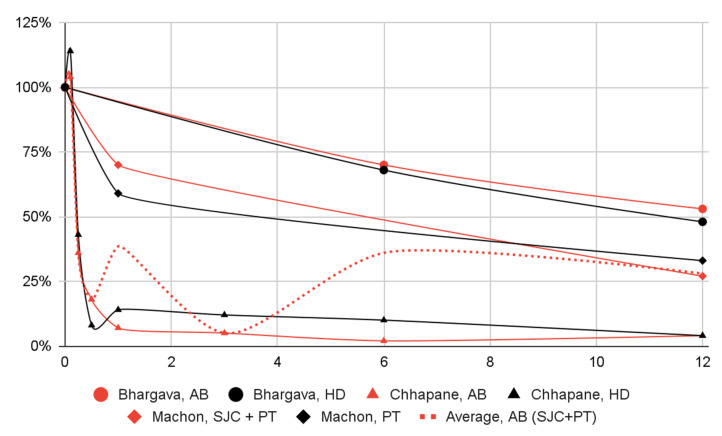
Change over time (in months) in articular pain severity (percentage of baseline) by patient group.

Significant decreases in: (1) the number of subjects with dislocation episodes; (2) the range of mandibular mobility; (3) and articular pain in most groups of patients were observed one month after the intervention. Dislocation episodes presumably subside immediately after blood administration. Data from a single study suggest that joint pain increases slightly three days after injection and is markedly relieved after one week. The effect in all domains persisted for at least a year, albeit further observations have not been conducted (Figure 3, Figure 4, Figure 5 and Figure 6, Table 8).

After 2–3 months, intracapsular and pericapsular administration of AB was better at reducing the number of dislocation episodes than an analogous intervention with hypertonic dextrose instead of blood (1 study, 32 patients, RR = 0.33, OR = 0.29) or without blood injection (1 study; 140 patients; insufficient data for quantification). However, no difference in outcomes was observed between the intracavitary and pericapsular administration of blood and an analogous intervention without intracapsular injection (2 studies; 70 patients in total; RR = 1, OR = 1). In one study, the same effect in the study and control groups persisted until the end of the one-year follow-up. In the pericapsular-only group of the second study, about a quarter of the patients initially treated had relapsed by six months compared to no relapse in the intracavitary and pericapsular injection group.

The results of the study by Chhapane et al. are outliers in the domains of resolving dislocation episodes and articular pain, which may be due to the small sample size.

## 4. Discussion

The mechanism of action of mandibular hypermobility therapy with intra-articular blood transfusions has not been fully elucidated. Despite obvious concerns, none of the screened abstracts mentioned ankylosis resulting from the administration of AB into or around the TMJ capsule. It is assumed that the limitation of mobility in the TMJ is achieved due to fibrosis, which was found in an animal model [13,14,15]. The lack of the articular surfaces’ cartilage disruption and leaving the articular disc intact presumably protect against ankylosis [12]. Moreover, in the course of AB therapy, the immobilization of the TMJs is partial and short-term or not implemented at all [7,9,42,43,44,49,51,52,55,56].

In a study of 16 pigs, injection of AB into the joint cavity (4 mL) and around the capsule (1 mL) has been shown to cause fibrosis of the retrodiscal ligaments in approximately 80% of individuals and less-marked fibrosis of the lateral portions of the capsule in over 50% of individuals [15]. The blood injection was preceded by rinsing the joint cavity with approximately 5 mL of Ringer’s solution, and postoperative immobilization was not applied [15]. The opposite joints of the same subjects underwent an intervention that differed by administering saline instead of blood [15]. In the control group, there were no signs of fibrosis within the discussed structures [15]. In another study, AB was injected into and around the TMJs of seven rabbits and the joints were immobilized for 2 days [14]. In the microscopic examination, only fibrosis was observed, without any structural changes within the TMJs [14]. Despite the very poor research material, it can therefore be suspected that the fibrosis of the retrodiscal tissues is primarily responsible for the reduced mobility of the mandible.

### 4.1. General Interpretation of the Results

#### 4.1.1. Dislocation Episodes

Given the modest amount of evidence from randomized controlled trials, the available studies clearly support the effectiveness of injecting AB into TMJs for the treatment of mandibular hypermobility. Synthesized studies indicate remission of dislocation episodes in most patients after the first administration. Applications may be reapplied until dislocation recurrences cease. There are currently no unified administration interval protocols. Most authors repeat the intervention in the event of redislocation and do so until the patient is successful or refuses. Non-respondents are usually offered more invasive therapies, but there are no unified protocols for the selection of surgical methods, which may be the subject of future research.

#### 4.1.2. Mandibular Mobility and Articular Pain

Within 1–12 months of the intervention, mandibular abduction was reduced to 80–90% of the initial value in randomized clinical trials [7,42,45,53,54,56]. In the same period, the articular pain values were inhomogeneous, but each time they were at least 30% lower than the initial value [7,8,45]. Due to the lack of longer follow-up, it is not known whether these treatment effects persist. In the treatment of temporomandibular disorders, in addition to unprocessed blood, centrifuged blood products such as platelet-rich plasma or injection fibrin are also used [20,21,58,59]. In simple terms, the indication for their administration is a painful limitation of mobility in the joint, and the effect of the therapy is pain relief and increased abduction [58,60,61,62,63].

#### 4.1.3. Autologous Blood Injections Compared to Hypertonic Dextrose Injections

The allegedly superior efficacy of blood to hypertonic dextrose in dislocation relief is based on only one study of 32 patients [8]. Both therapies are probably highly effective, however, based on the collected material, no further conclusions can be drawn and this issue undoubtedly requires further research.

#### 4.1.4. Peri- and Intracapsular Injections versus Pericapsular Injection Alone

Over a period of 1–3 months, pericapsular injections alone seemed to be comparable in resolving dislocations to pericapsular injections conducted along with intracavitary ones [45,56]. Relapses were observed in the group without intracapsular deposition [45]. Differences between both peri- and intracapsular injections versus pericapsular injections alone were not investigated in the previously discussed animal studies [14,15]. Considering the fibrosis of the retrodiscal ligaments observed in pigs, the question of the effectiveness of AB deposition in the retrodiscal area alone should be answered in future studies [15].

### 4.2. Limitations of the Evidence

Six of the eight randomized controlled trials raised some concerns about the risk of bias. A study by Machon et al. (2018) consisted of a single intervention, and all other randomized controlled trials allowed repeated injections until successful, subject to patient consent. The different number of repetitions of the intervention makes it difficult to draw conclusions about the effectiveness of a single administration. The inhomogeneity of the control groups makes it difficult to compare AB injections for TMJs with other interventions. In the article by Chhapane et al. inconsistencies were noted between the description and the figures of the mandibular abduction, suggesting a simple typing error. This could not be verified as an attempt to contact the authors was unsuccessful. Therefore, these data were not included in the quantitative analysis.

### 4.3. Limitations of the Review Processes

Search queries contained only English keywords, which excluded papers without even titles translated into this language.

## 5. Conclusions

Injections of autologous blood into the temporomandibular joints were effective in preventing further TMJ dislocation episodes in 75–94% of patients. Mouth opening was reduced by 10–20% and the articular pain subsided noticeably. Despite the unclear mechanism of action, no cases of post-interventional ankylosis were identified.

## Figures and Tables

**Table 1:** Eligibility criteria.

	Inclusion Criteria	Exclusion Criteria
Problem	Mandibular hypermobility	Cadaver and animal studies
Intervention	Unprocessed blood intra- or pericavitary injection(s)	More invasive interventions, such as arthroscopy or open surgery
Comparison	Arthrocentesis, placebo injection, hypertonic dextrose prolotherapy, immobilization, and physiotherapy	As above
Outcomes	Frequency or presence of dislocation episodes, range of mandibular mobility, articular pain	Not applicable
Timeframe	Any	Not applicable
Settings	Primary studies	Case reports and series of up to three cases

**Table 2:** Study characteristics.

First author	Patients	Injection Site	Volume	Cointervention	Repetitions	Control	Evidence Level
Bhargava [7]	60	Bilateral	3 mL	Immobilization	1–4	HD	2
Chhapane [8]	32	Unilateral or bilateral	3 mL	None	1–2	HD	2
Sharma [12]	30	14 unilateral16 bilateral	3 mL	Arthrocentesis	1–3	None	4
Pandey [9]	20	Bilateral	3 mL	Immobilization	1	HD	3
Shah [40]	5	N/S	5 mL	Arthrocentesis	1	None	4
Ertas [41]	300	Bilateral	5 mL	None	1	Placebo	2
Amer [42]	140	Bilateral	3 mL	Arthrocentesis + immobilization	1–2	Arthrocentesis alone	2
Bukhari [43]	80	Bilateral	3 mL	Immobilization	1	Intracapsular injections alone	3
Gagnani [44]	19	4 unilateral15 bilateral	3 mL	Immobilization	1–2	None	4
Machon (2018) [45]	40	Unilateral	3 mL	None	1	Intracapsular injections alone	2
Yoshida [46]	21	13 unilateral8 bilateral	N/S	N/S	1–3	None	4
Patel [47]	10	Bilateral	3 mL	Arthrocentesis	1	None	4
Ahmed [48]	11	Bilateral	4 mL	Arthrocentesis	1	None	4
Coser [49]	11	Bilateral	3 mL	Arthrocentesis + immobilization	1–2	None	4
Oshiro [50]	14	Unilateral	5 mL	None	1	None	4
Bayoumi [51]	15	Bilateral	3 mL	Arthrocentesis + immobilization	1	None	4
Candirli (2013) [52]	17	5 unilateral12 bilateral	5 mL	Immobilization	1–2	None	4
Hegab [53]	48	Bilateral	5 mL	Arthrocentesis	1–3	AB + Immobilization; immobilization alone	2
Triantafillidou [54]	40	2 unilateral23 bilateral	3 mL	None	1–4	Physiotherapy	2
Candirli (2012) [55]	14	8 unilateral6 bilateral	5 mL	Immobilization	1	None	4
Daif [56]	30	Bilateral	3 mL	Arthrocentesis + immobilization	1	Intracapsular injections alone	2
Machon (2009) [57]	25	Bilateral	3 mL	Arthrocentesis	1–3	None	4

HD–Hypertonic dextrose; AB–Autologous blood; N/S–not specified.

**Table 3:** Risk of bias in studies.

Study	Randomization	Deviations	Missing Outcome	Measurement	Selection	Overall
Bhargava	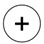	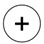	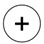	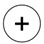	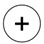	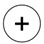
Chhapane	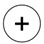	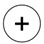	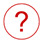	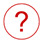	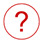	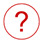
Ertas	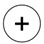	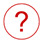	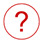	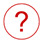	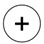	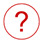
Amer	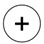	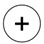	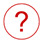	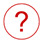	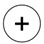	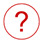
Machon	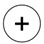	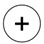	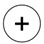	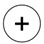	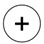	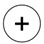
Hegab	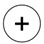	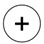	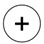	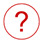	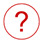	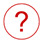
Triantafillidou	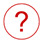	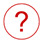	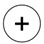	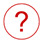	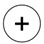	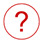
Daif	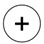	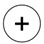	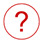	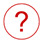	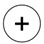	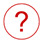

+—low risk of bias; ?—some concerns.

**Table 4:** Study population and reported treatment effects.

Study	Population (Mean Age, Male/Female)	Dislocation Episodes	Mandibular Abduction	Articular Pain	Other
Bhargava	29.2 ± 8.5 33/27	No	Yes	Yes	None
Chhapane	36.6 ± 8.8 14/18	Yes	Incorrect data *	Yes	Acoustic symptoms
Ertas	31.0 ± 5.5 82/218	No	No	No	Craniomandibular index
Amer	46.6 ± 8.5 and 49.3 ± 6.3 ** 56/84	Yes	Yes	No	Acoustic symptoms, satisfaction
Machon	29.9 *** 3/37	Yes	Yes	Yes	None
Hegab	33 *** 11/37	Missing data	Yes	No	None
Triantafillidou	33.5 and 34.3 **^,^*** 9/31	No	Yes	No	Acoustic symptoms
Daif	34 *** 12/18	Yes	Yes	No	None

* Text information contradicts numerical values. ** Data for individual patient groups. *** No standard deviation provided.

**Table 5:** Patients presenting dislocation episodes.

Patient Group	Group Size	Initial Value	2 Weeks	1 Month	2 Months	3 Months	6 Months	12 Months
Chhapane, AB	16	16 100%	N/S	N/S	N/S	1 6%	N/S	0 0%
Chhapane, HD	16	16 100%	N/S	N/S	N/S	3 19%	N/S	0 0%
Amer, AB	70	70 100%	N/S	N/S	18 * 25%	N/S	N/S	N/S
Amer, non-AB	70	70 100%	N/S	N/S	28 * 40%	N/S	N/S	N/S
Machon, SJC + PT	20	20 100%	N/S	210%	N/S	4 20%	4 20%	4 20%
Machon, PT	20	20 100%	N/S	0 0%	N/S	5 25%	9 45%	9 45%
Daif, SJC + PT	15	15 100%	4 27%	2 13%	N/S	2 13%	2 13%	2 13%
Daif, PT	15	15 100%	2 13%	1 7%	N/S	1 7%	1 7%	1 7%

AB—autologous blood, N/S—not specified, HD—hypertonic dextrose, SJC—superior joint compartment, PT—pericapsular tissues, * values estimated by multiplying the average number of dislocation episodes and the number of patients.

**Table 6:** Mandibular abduction in millimeters.

Patient Group	Group Size	Initial Value	1 Month	2 Months	3 Months	6 Months	12 Months
Bhargava, AB	30	43 100%	N/S	N/S	N/S	39 91%	38 88%
Bhargava, HD	30	43 100%	N/S	N/S	N/S	39 91%	38 88%
Amer, AB	70	47 100%	N/S	40 85%	N/S	N/S	N/S
Amer, non-AB	70	44 100%	N/S	40 91%	N/S	N/S	N/S
Machon, SJC + PT	20	N/S 100%	N/S	N/S	N/S	N/S	N/S 86%
Machon, PT	20	N/S 100%	N/S	N/S	N/S	N/S	N/S 88%
Hegab, AB	16	50 100%	40 80%	N/S	41 82%	41 82%	42 84%
Hegab, IMF	16	51 100%	40 78%	N/S	41 80%	41 80%	42 82%
Hegab, AB + IMF	16	51 100%	37 73%	N/S	38 75%	3976%	40 78%
Triantafillidou, AB	25	50 100%	N/S	N/S	43 86%	N/S	N/S
Triantafillidou, P	15	50 100%	N/S	N/S	49 98%	N/S	N/S
Daif, SJC + PT	15	41 * 100%	N/S	N/S	N/S	N/S	37 90%
Daif, PT	15	41 * 100%	N/S	N/S	N/S	N/S	35 85%

AB—autologous blood, N/S—not specified, HD—hypertonic dextrose, SJC—superior joint compartment, PT—pericapsular tissues, IMF—intermaxillary fixation, P—physiotherapy, * average calculated for all patients.

**Table 7:** Articular pain in visual analog scale.

Patient Group	Group Size	Initial Value	3 Days	1 Week	2 Weeks	1 Month	3 Months	6 Months	12 Months
Bhargava, AB	30	8.9 100%	N/S	N/S	N/S	N/S	N/S	6.2 70%	4.7 53%
Bhargava, HD	30	8.4 100%	N/S	N/S	N/S	N/S	N/S	5.7 68%	4.0 48%
Chhapane, AB	16	5.5 100%	5.7 104%	2.0 36%	1.0 18%	0.4 7%	0.3 5%	0.1 2%	0.2 4%
Chhapane, HD	16	5.1 100%	5.8 114%	2.2 43%	0.4 8%	0.7 14%	0.6 12%	0.5 10%	0.2 4%
Machon, SJC + PT	20	4.4 100%	N/S	N/S	N/S	3.1 70%	N/S	N/S	1.2 27%
Machon, PT	20	4.2 100%	N/S	N/S	N/S	2.5 59%	N/S	N/S	1.4 33%

AB—autologous blood, N/S—not specified, HD—hypertonic dextrose, SJC—superior joint compartment, PT—pericapsular tissues.

**Table 8:** Summary of findings in the groups receiving intra- and pericapsular autologous blood injections. In the case of multiple results available in the period of 1–6 months of observation, later values were used for calculations.

Outcome Domain	Number of Studies Presenting Outcomes after 1–6 Months	Total Number of Patients	Weighted Average of the Effect after 2–6 Months	Standard Deviation	Risk of Bias in Studies
Dislocation episodes	4	121	20% of the initial number of patients presenting dislocation episodes	8%	Some concerns
Mandibular abduction	4	176	86% of initial mandibular abduction	4%	Some concerns
Articular pain in visual analogue scale	3	66	54% of initial severity of pain	39%	Some concerns

## Data Availability

All collected data are included in the content of this article. PROSPERO registration number: CRD42023447156.
